# Model-independent particle species disentanglement by X-ray cross-correlation scattering

**DOI:** 10.1038/srep45618

**Published:** 2017-04-04

**Authors:** B. Pedrini, A. Menzel, V. A. Guzenko, C. David, R. Abela, C. Gutt

**Affiliations:** 1 Paul Scherrer Institute, 5232 Villigen PSI, Switzerland; 2 Department Physik, Naturwissenschaftlich-Technische Fakultät, Universität Siegen, 57068, Siegen, Germany

## Abstract

Mixtures of different particle species are often investigated using the angular averages of the scattered X-ray intensity. The number of species is deduced by singular value decomposition methods. The full disentanglement of the data into per-species contributions requires additional knowledge about the system under investigation. We propose to exploit higher-order angular X-ray intensity correlations with a new computational protocol, which we apply to synchrotron data from two-species mixtures of two-dimensional static test nanoparticles. Without any other information besides the correlations, we demonstrate the assessment of particle species concentrations in the measured data sets, as well as the full *ab initio* reconstruction of both particle structures. The concept extends straightforwardly to more species and to the three-dimensional case, whereby the practical application will require the measurements to be performed at an X-ray free electron laser.

Structural features of particles whose orientation cannot be controlled experimentally can be studied by evaluating the angular mean of the X-ray scattering signal, averaged on a large ensemble of random particle configurations. For proteins in solution, for example, the signal at small angles encodes enough information to model molecular shapes with a precision of few nanometers^1^,^2^,^3^. It is often of interest to extend the perspective from pure samples to mixtures of more than one particle species or, similarly, of more configurations of the same particle. In case of dynamical studies, for instance, it is implicit that a number of data sets are collected at different time points, corresponding to different mixture compositions^4^,^5^,^6^,^7^,^8^,^9^,^10^. The angular means of the X-ray intensities can then be processed using the singular value decomposition algorithm^11^,^12^,^13^ (SVD), which serves as a noise filter and yields the number the particle species present in the system. Unfortunately, further processing to establish the particle species populations or their respective scattering intensities and structures needs additional prior knowledge. For time-resolved studies, for example, one can fit the data to a known kinetics model^11^,^14^.

For the single-species case, already in 1977 Zvi Kam suggested to exploit higher-order angular correlations^15^ of the scattered X-ray intensity, which contain a wealth of additional structural information with respect to the angular means. In the subsequent decades, the idea was further developed for other applications^16^,^17^,^18^, but consistent interest in the X-ray community emerged only recently with the advent of X-ray free electron lasers^19^ (XFELs). The investigations on disordered systems reported by Wochner and coworkers^20^ in 2009 were followed by a cascade of related theoretical^21^,^22^,^23^,^24^,^25^,^26^ and experimental proof-of-concept publications^27^,^28^,^29^. In contrast to the angular means, proper evaluation of the higher-order correlations requires that the randomly oriented particles do not rotate within an X-ray exposure. In addition, the figure of merit for the achievable signal-to-noise is given by the scattering strength per exposure of a single particle^30^, which is generally extremely weak for scientifically relevant samples such as macromolecules. The ultrashort length and ultrahigh intensity of XFEL pulses are precisely the two features necessary to overcome these obstacles.

In this paper, we investigate the application of the framework of higher-order angular correlations to the case of multiple particle species. This path was already followed by the authors of ref. 31 who attempted to disentangle the experimental correlations from mixtures into single-particle contributions. However, the proposed method relies fully on the prior knowledge of the species concentrations in all data sets, which represents a strong limitation for most applications. We present here a new computational protocol that achieves the disentanglement without any *a priori* knowledge. All intermediate steps are well-established procedures with the exception of the key step, for which the underlying concept is condensed in Eqs (13) and (20) for the two- respectively three-dimensional case. These equations imply that the angular 2-point intensity correlations contain intrinsically the information necessary to perform the disentanglement. The protocol allows obtaining, in a model-independent fashion, the concentrations of the species as well as their individual structures.

We describe the protocol in the two-dimensional case, and illustrate in detail the results of the application of the protocol to experimental data measured at a synchrotron source on static test samples with two particle species. We further address the generalization to more species, the implications for the three-dimensional case, and the possible implementation at XFELs.

## X-ray intensity correlations based disentanglement in two dimensions

We consider the two-dimensional (2D) case, i.e. 2D particles on a plane perpendicular to the X-ray beam direction. For each of the *n*_s_ particle species, labeled with *a*, we denote by


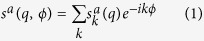


the diffraction image generated by a single particle, where (*q, ϕ*) are polar coordinates in reciprocal space.

The input experimental data are arranged in a number *n*_d_ of different data sets with label *r*. A data set consists of a large number of X-ray diffraction images 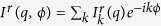 from randomly oriented and positioned particles in defined experimental conditions, which yield to data set specific average numbers of illuminated particles *N*^*a,r*^ of species *a*. For each data set *r*, the angular averages, here denoted as 1-point correlations, are given by


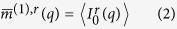


where 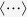 denotes the average over all the images in the data set. Similarly, the 2-point correlations are defined in terms of their angular Fourier components as


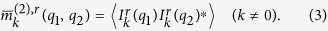


The single-particle correlations of each species are defined as


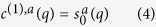


and


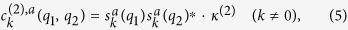


with *κ*^(2)^ a constant X-ray beam shape factor^27^. For known single-particle correlations, these equations can be solved for the coefficients 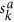 following an established protocol^27^, which finally yields the 2D charge density of the particle.

For mixtures with *n*_s_ different species the single-particle 1-point correlations are related to the experimental 1-point correlations by the linear equations


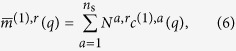


while for the 2-point correlations we have


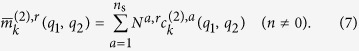


Equality is approached in the limit of an infinite number of measured images, or, equivalently, of sampling all particle configurations. Both equalities (6) and (7) are a straightforward generalization of the single species case^21^,^27^,^31^. The experimental correlations are measured at *n*_*q*_ discrete values of momentum transfers *q*. Therefore, the 1-point correlations can be grouped into an *n*_*q*_ × 1 dimensional matrix, while for each Fourier order *k* the 2-point correlations can be grouped into an *n*_*q*_ × *n*_*q*_ dimensional Hermitian matrix. To simplify the notation, all experimental correlations of data set *r* are furthermore rearranged into a column 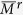 of a *n*_m_ × *n*_d_-dimensional matrix 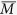 (see Supplementary Figure S1). *n*_m_ is the total number of measured correlations, given by sum of the number of 1-point correlations *n*_*q*_ and the number of 2-point correlations 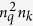, with *n*_*k*_ the number of considered Fourier components of the 2-point correlations. Similarly, the single-particle correlations of species *a* are rearranged into a column *C*^*a*^ of a *n*_m_ × *n*_s_-dimensional matrix *C*. With this notation, equations (6) and (7) can be recast in compact matrix form as


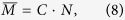


where *N* is the *n*_s_ × *n*_d_-dimensional population matrix with entries *N*^*a,r*^.

**Figure 1:**
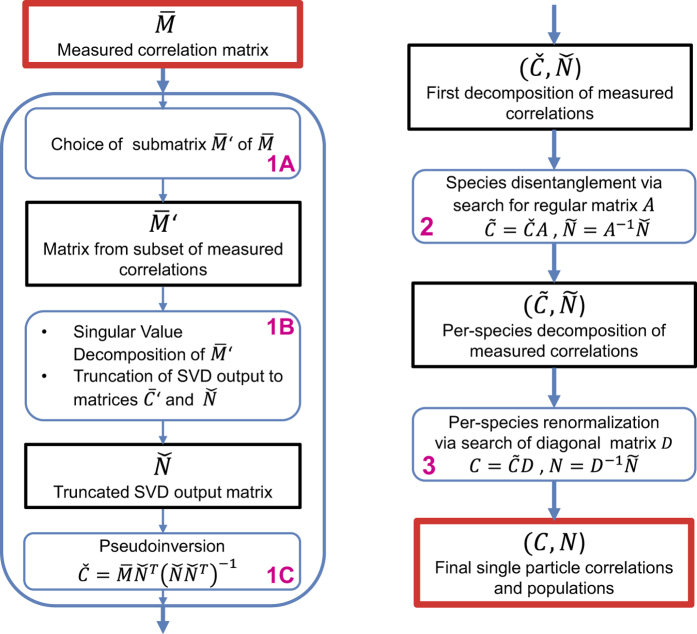
Algorithm for species disentanglement. Graphical illustration of the three-step algorithm used to determine the final single-particle correlations *C* and populations *N* from the experimental correlations 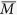. Step 1: SVD-based determination of the pair 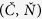; 1A consists in defining a subset of experimental correlations, 1B is the singular value decomposition step thereof, followed by the truncation to the significant non-vanishing singular values, and 1C is the pseudoinversion of 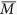 with 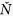. Step 2: particle species disentanglement mediated by the regular matrix *A* via 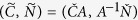. Step 3: per-species renormalization of correlations and populations mediated by the diagonal matrix *D* via 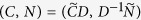.

Thus, disentangling the data into contributions from the individual species means finding the right matrices *C* and *N* which fulfill Eq. (8). In the framework considered in ref. 31, the population matrix *N* and therefore the number of species *n*_s_ were assumed to be known. The single-particle correlations were then computed straightforwardly by the matrix inversion 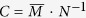. Without these assumptions, however, the problem is far from being trivial because Eq. (8) has not a unique solution. Without knowledge of *n*_s_, not even the dimensions of the matrices *N* and *C* are defined. We propose here a new algorithm which serves to uniquely determine the right solution without any prior knowledge. The algorithm is illustrated in Fig. 1 and consists of three subsequent steps.

In the step 1, we establish the number of species *n*_s_ and a pair of matrices 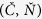 of dimensions *n*_m_ × *n*_s_ and *n*_s_ × *n*_d_ such that


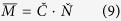


within small errors. Specifically, we follow a bootstrap approach by defining different matrices 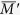, obtained choosing different rows from 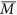 (step 1A), and applying the SVD (step 1B) as in Eq. (23) in the Methods. For each choice the decomposition is different. However, the expected self consistency of the data must result in the same number of meaningful singular values to be retained, which corresponds to the number of species *n*_s_. Furthermore, the populations 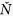 obtained after truncation to *n*_s_ species must all be pairwise equivalent, meaning that for any pair 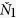 and 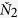 resulting from different decompositions the relationship 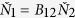 must hold upon small errors for some regular *n*_s_-dimensional matrix *B*_12_. If self-consistency is verified, we choose 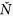 as the outcome of one of the decompositions, and define 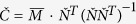 via pseudoinversion^12^ (step 1C).

The key procedure of disentangling the individual species takes place in step 2. We seek for a regular *n*_s_-dimensional matrix *A* which mediates the transformation


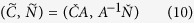


that obviously preserves (9),


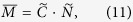


in such a way that the correlations encoded in each column 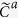 of the matrix 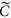 is compatible with coming from a single particle. We impose this condition by making use of information which is intrinsically contained in the 2-point correlations. Indeed, Eq. (5) implies that for each column *a* the corresponding 2-point correlation matrices of each Fourier order *k* must be of the form


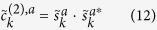


for some column vector 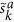. This requirement is equivalent to


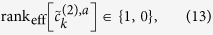


where the effective rank rank_eff_ is the number of significant non-vanishing eigenvalues. The exceptional case 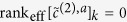 occurs if and only if the species lacks the 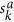-component in its diffraction pattern. To determine the matrix *A* we therefore apply a procedure that analyzes the behavior of the eigenvalues of the Hermitian matrices 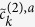, which is described in the Methods and explained in detail in the Results section for the special case *n*_s_ = 2.

Step 3 of the procedure is the same per-species renormalization of the Fourier components as in the single-species case (see Methods). The transformation is of the form


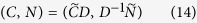


with *D* a diagonal matrix, yielding final single-particle correlations and populations in agreement with the experimental data by


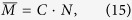


upon small errors.

## Results from the proof-of-concept experiment

**Figure 2:**
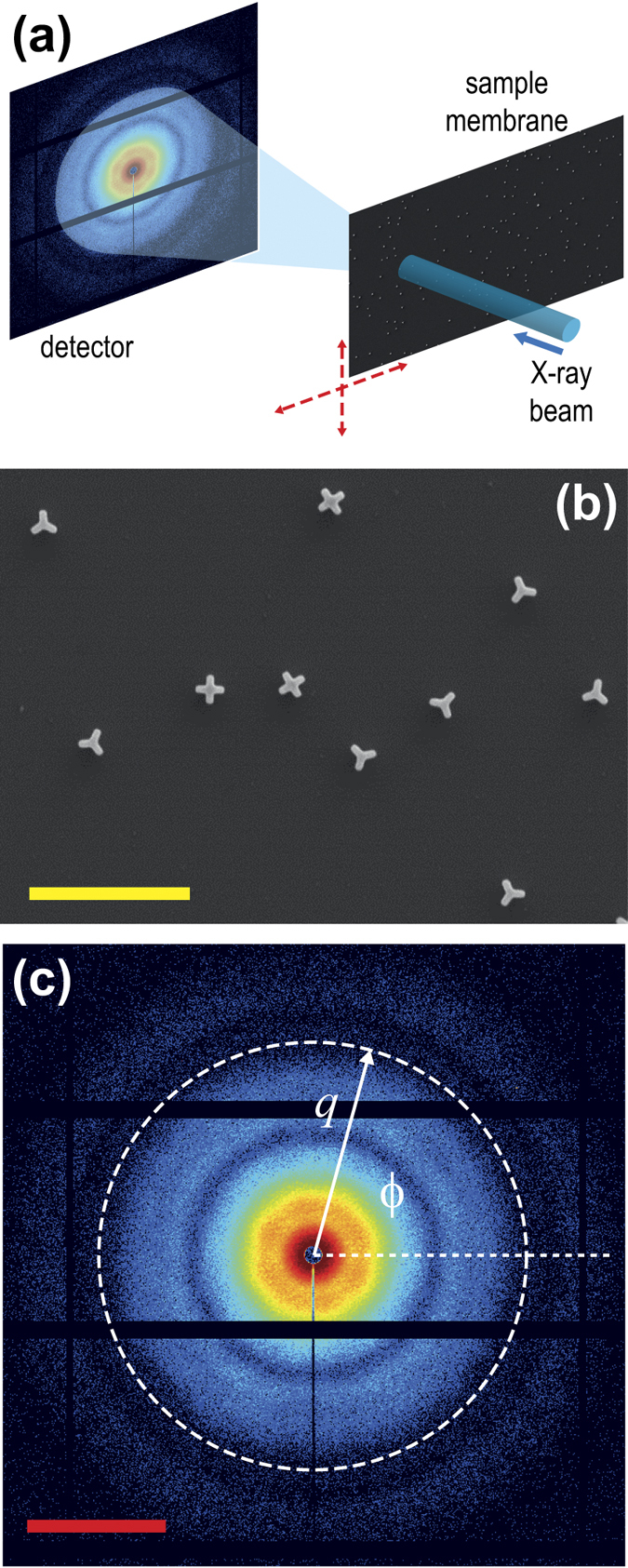
Data acquisition and cross-correlation calculations. (**a**) Experimental setup of the proof-of-concept experiment: the membrane is scanned through the beam, and a large number of diffraction images is acquired from different membrane positions. (**b**) SEM image of a portion of one of the six sample membranes measured during the experiment, which shows the two species with four- respectively three-fold symmetry. In this sample, the concentration ratio was 0.4/0.6. The yellow bar in the lower left corner corresponds to 2 *μ*m. (**c**) Example of an acquired diffraction image *I(q, ϕ*), with graphical illustration of the radial and azimuthal reciprocal space coordinates *q* and *ϕ*. The red scale bar corresponds to 0.1 nm^−1^.

For the proof-of-concept experiment, we considered the two-species case (*n*_s_ = 2) with four- and three-fold symmetric 2D particles (Fig. 2(b)). The high symmetry of the particles used in the experiment did not represent a simplification but rather a complication, since the number of non-vanishing Fourier components of the 2-point correlations is reduced. Six different samples were measured. Each sample consisted of a thin membrane with a large number of gold nanostructures, anchored in random position and random orientation with respect to the axis perpendicular to the membrane (Fig. 2(b)). The total average surface density was the same in all samples, but the relative concentration was sample specific. The six sample membranes were scanned with the X-ray beam perpendicular to the surface, and thousands of snapshot diffraction images were recorded by illuminating each time different small areas including tens of nanostructures in random configuration (Fig. 2(a)). This resulted in six data sets (*n*_d_ = 6) characterized by well-defined species populations. Figure 2(c) shows an example of a collected diffraction image. The experimental correlations were calculated as described in the Methods, retaining Fourier coefficients of the 2-point correlation up to order *k* = 40, and were rearranged in the matrix 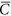, with each of the six columns *r* = 1..6 corresponding to a data set (see Supplementary Figure S1).

**Figure 3:**
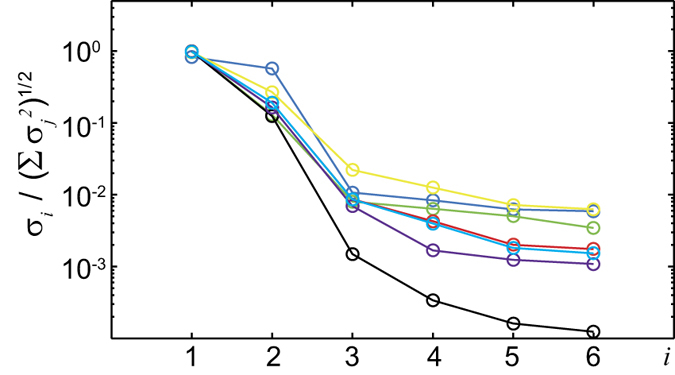
Singular values. Singular values resulting from the SVD applied to seven different subsets *M*′ of experimental 1- and 2-point correlations. The singular values *σ*_1_ to *σ*_6_ are shown in decreasing order on the y-axis, rescaled according to 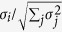.

Within step 1 of our protocol, we applied the SVD to seven correlation subsets 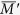 of the measured correlations 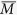. The resulting singular values are plotted in decreasing order in Fig. 3. We observe a marked drop in magnitude between the second and third singular value, pointing towards the presence of *n*_s_ = 2 particle species in the mixtures. This is confirmed by the fact that only by retaining two singular values the decompositions are mutually self-consistent. The population matrix 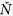 resulting from the decomposition performed on all measured correlations was used to calculate via pseudoinversion the matrix 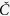.

**Figure 4:**
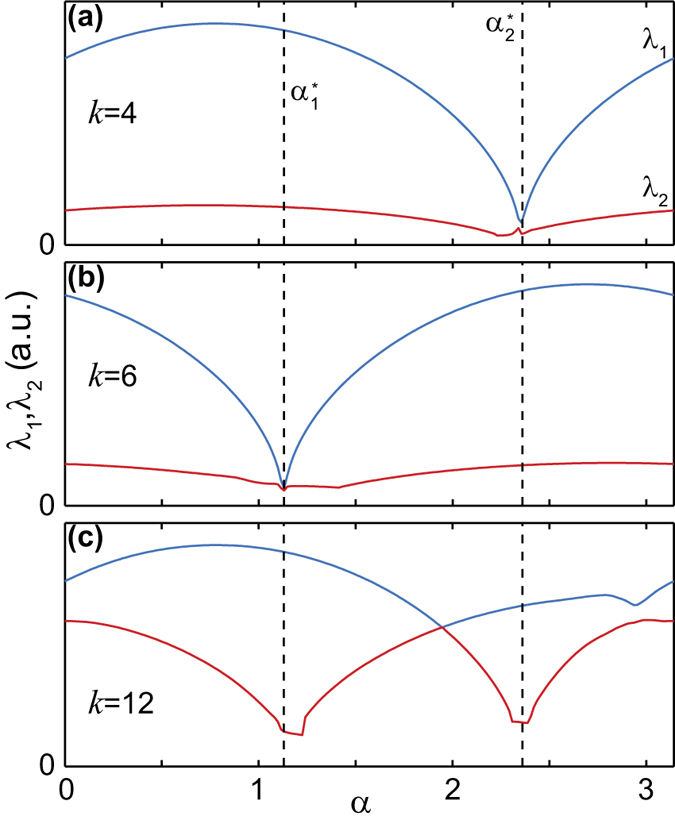
2-point correlation matrix eigenvalues. The two largest eigenvalues *λ*_1_ (blue graphs) and *λ*_2_ (red graphs) of the 2-point correlation matrices 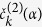 (see text) are displayed as a function of the parameter *α* for the Fourier coefficient orders *k* = 4, 6, 12. The vertical dashed lines mark the disentangling points 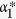 and 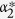.

In step 2, the 2-dimensional invertible matrix *A* appearing in Eq. (10) and leading to Eq. (13) was determined as follows. From each column *a* = 1, 2 of 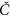 we extracted the 2-point correlation matrices 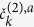 (see Supplemntary Figure S1), defined the 2-point correlation matrices


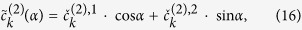


and studied the behavior of the two largest eigenvalues as a function of the parameter *α*, shown in Fig. 4 for *n* = 4, 6, 12. Indicated are the two values 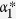 and 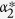 of the parameter at which the effective rank of the matrix 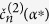 is either 1 or 0. For *k* = 12, |*λ*_1_| and |*λ*_2_| vary as a function of *α*, a clear signature of the contribution from two distinct particle species. The requirement 
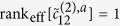
 is fulfilled at the local minima of |*λ*_2_|. Differently, for *k* = 4, 6 |*λ*_2_| is small and almost constant, which is the signature of resulting from noise. Only a single species contributes, and the disentangling points are located at the local minima of |*λ*_1_|, where 
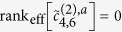
. As expected for consistency, 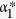 and 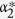 take the same value for all orders *k*. The matrix *A* is defined as


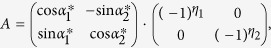


where the signs 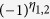 are to be chosen such that 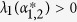, and is used to set the matrix pair 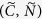 according to (10). This ensures that the 2-point correlations in the two columns of 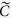, given by 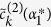 and 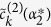, fulfill Eq. (13).

**Figure 5:**
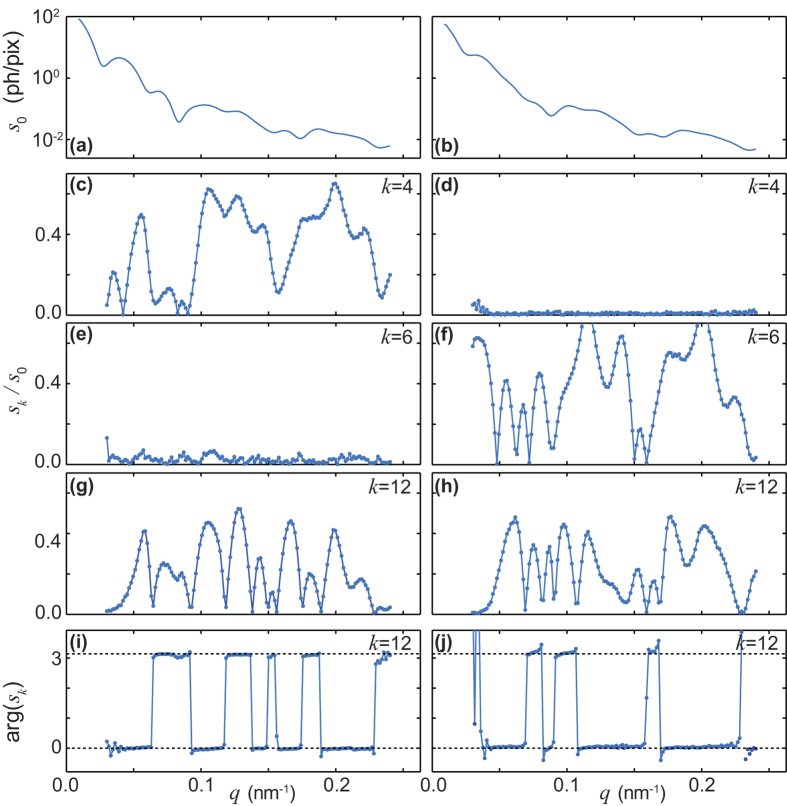
Single-particle diffraction pattern Fourier coefficients. The left and right columns are for the four- respectively three-fold symmetric particle species. The plots show the *ϕ*-Fourier components *s*_*k*_(*q*) of the single-particle diffraction image *S(q, ϕ*). (**a**,**b**) *s*_0_ displayed as a function of the momentum transfer *q*. (**c**–**h**) Amplitudes of *s*_*k*_ as a function of *q*, after normalization with *s*_0_, for *k* = 4, 6, 12. (**i**,**j**) Phases of *s*_12_ as a function of *q*.

**Figure 6:**
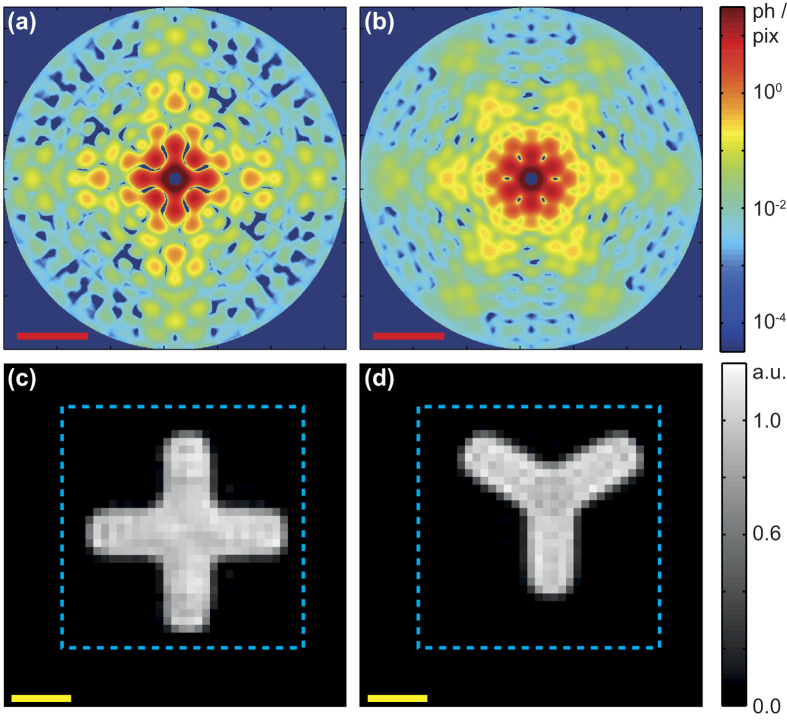
Two-dimensional structures. The left and right columns are for the four- respectively three-fold symmetric particle species. (**a**,**b**) Single-particle diffraction patterns *s(q, ϕ*) in reciprocal space. The color coding corresponds to a logarthimic scale for the number of diffracted photons per detector pixel. The red scale bars correspond to 0.1 nm^−1^. (**c**,**d**) Two-dimensional single-particle charge density, calculated from (**a**,**b**), respectively, by applying a phase retrieval algorithm. The dashed box in cyan corresponds to the imposed compact support. The yellow scale bars correspond to 100 nm.

In the final step 3, we renormalized single-particle correlations and populations to final values represented by the matrices (*C, N*) with Eq. (14), and in parallel determined the single particle diffraction pattern of both particle species (see Methods). Examples of the Fourier coefficients 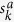 for both particle species are shown in Fig. 5. The coefficients 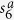 for *a* = 1 and 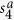 for *a* = 2 turn out to vanish, which is in line with the four- and three-fold symmetry of the two species. The patterns *s(q, ϕ*) are shown in Fig. 6(a,b), from which we reconstructed the 2D charge density by phase retrieval. The obtained shapes are shown in Fig. 6(c,d) and agree very well with the shapes observed in the SEM images. The final population matrix is


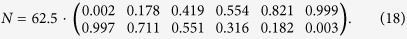


The first and second rows of (18) correspond to the four- and three-fold symmetric particles, respectively, and the six columns are related to the six recorded data sets. The relative populations agree reasonably with the nominal values reported in the Methods. We attribute the discrepancies to slight differences in the nanostructure height in the different samples.

To ensure that the obtained results are not biased by including the pure data sets (*r* = 1, 6) in the analysis, we applied the whole protocol to the other four mixed data sets, and obtained, within the experimental uncertainty, the same particle populations and the same single particle structures.

## Discussion

The results from the proof-of-concept experiment demonstrate that the proposed protocol is effectively capable of full disentanglement of the two particle species from data acquired on unknown admixtures. For more than two particle species, the computational part is obviously more elaborated but conceptually the same (see Methods). The crucial aspect is to exploit the 2-point correlations with Eq. (13). The conceptual and experimental boundary conditions are almost the same as for the single-species case: The diffraction images must be snapshots from particles in random position and in random orientation with respect to the X-ray beam axis. Additionally, each species must exhibit non-vanishing 2-point correlations, i.e. cannot be rotation symmetric. In practice, this is assessed by verifying that the number of relevant SVD singular values from 1-point correlations and from 2-point correlations are the same.

We can take the point of view of the reported measurements exemplifying a time-resolved experiment which monitors a structural process taking place in the liquid or gas phase. Each image is an X-ray snapshot of a particle arrangement at a given time delay after the transformation has been initiated. Different data sets correspond to different evolution times. The reaction consists in a four-fold symmetric particle transforming itself with a certain transition rate into a three-fold symmetric one (or vice-versa). The transformation is instantaneous in the sense that the probability of observing any particle in a transition state is negligible. The evaluation is not biased by any assumption about time-evolution and/or stochiometry of the process. For instance, from Eq. (18) we deduce that the total number of particles *N*^1,*r*^ + *N*^2,*r*^ in each data set *r* is almost constant, pointing towards a 1:1 stochiometry.

We have addressed the 2D case because it is more suitable for a proof-of-concept experiment. However, the relevant application is in three dimensions (3D), meaning that the particles have full *SO*(3) rotational freedom, as well as three instead of two translational degrees of freedom. The experimental 1- and 2-point angular correlations are calculated in the same way as for the 2D case from a large set of multi-particle X-ray diffraction images, with the only difference being that the 2-point correlations in the 3D version are obtained by inverse Fourier transformation followed by Legendre transformation of the definition of Eq. (3), i. e. 
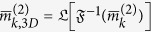
15. The 3D equivalent of Eq. (5) then becomes^15^,^17^


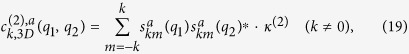


where 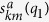 are the spherical harmonic components of the 3D diffraction intensity *I(q, θ, ϕ*). From Eq. (19) it follows that the 3D analog of Eq. (13) is


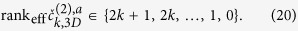


In the general case, 
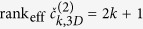
. A smaller value of rank_eff_ is distinctive of symmetries of the particle. For example, axial symmetry results in rank_eff_ = 1 for all even orders *k* because *s*_*km*_ ≡ 0 for *m* ≠ 0. Eq. (20) can be used to disentangle the data along the same lines described in the previous sections for the 2D case exploiting Eq. (13). We briefly describe the concept for the case of two particle species, both devoid of any symmetry. It is convenient to consider the lowest order *k* = 2, for which Eq. (20) becomes 
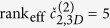
. The search of the disentangling parameter is performed analogously as shown in Fig. 4 but monitoring the behaviour of the six (or more) dominant eigenvalues of 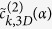, instead of 2 (or more). A disentangling point α^*^ is characterized by the sixth (and higher) eigenvalues dropping towards zero, as does the second eigenvalue in Fig. 4 for *k* = 12.

Scientifically interesting objects may be biological macromolecules or even smaller organic and inorganic molecules, showing structural dynamics among different conformations. For the experiments, the particles are most likely in the gas phase or in solution. Application of higher-order angular X-ray intensity correlation methods on these systems is favorable with respect to other approaches^32^ because it requires the control of the average number of particles exposed to the X-rays (species populations), but not of the number in each exposure. Furthermore, studying these systems requires the measurements to be performed at an XFEL. The two reasons are the same as in the case of a single particle species^30^. First, the particle tumbling timescales are in the picosecond to nanosecond regime, and the femtosecond XFEL pulses are short enough to freeze the rotational motion. Second, the particles may exhibit an extremely weak X-ray diffracting power, which is accentuated by the required short X-ray exposure times. The ultraintense and ultrashort XFEL pulses maximize the number of diffracted photons exploiting the diffract-before-destroy data collection mode^33^,^34^,^35^. However, even with XFELs the limited number of diffracted photons per particle remains the major limitation, which can be counteracted only by acquiring a sufficiently large number of diffraction images. The signal to noise ratio (SNR) that can be obtained in the 2-point angular correlations is here the figure of merit to evaluate the feasibility of the experiment. The authors of ref. 30 have proven that the SNR scales with the square root of the number of images used to calculate the correlations and is proportional to the number of scattered photon per particle at the considered resolution. Using realistic XFEL parameters (10^12^ 1.5 Å photons focused in a 100 nm spot^36^), the reported number of images necessary to achieve a SNR of 2 at 1 nm resolution on a typical biological macromolecule is 10^7^. With the frame acquisition rate of the order of 10^4^ frames per second, achievable at the European XFEL^37^ which is foreseen to start operation in 2017, a dataset could therefore be measured within less than an hour.

In summary, we have presented a new analysis protocol for X-ray diffraction data from diluted, disordered mixtures of different particle species. As demonstrated with the proof-of-concept experiment, a proper evaluation of the angular 2-point X-ray intensity correlations permits the problem to be disentangled into single-species sets of correlations and in parallel the species populations to be deduced, without relying on any information other than that contained in the X-ray data themselves. For practical applications, the obstacles are the same as for a single species and may be overcome in the close future thanks to the ultrashort and ultraintense X-ray pulses delivered by free electron lasers.

## Methods

### Samples

The samples were fabricated as described in refs 38 and 39. The gold nanoparticles were grown on a 200 nm thick Si_3_N_4_ membrane coated with a gold seed layer. Each nanoparticle’s orientation and position were selected randomly and mutually independently from uniform distributions over the [0, 2*π*] angle range and over the 800 × 800 *μ*m^2^ sample area, respectively. The only inter-particle correlation resulted from the requirement of a minimum distance between neighboring particles of 500 nm, imposed in order to avoid particle overlap. The measurements were performed on six different samples containing two different particle species with the following concentration ratios: 0.0/1.0, 0.2/0.8, 0.4/0.6, 0.6/0.4, 0.8/0.2 and 1.0/0.0. All samples had the same total average particle surface density of 10 particles per 100 *μ*m^2^ area.

### Beamline setup and data acquisition

The measurements were performed at the cSAXS beamline of the Swiss Light Source. The photon energy was 6.2 keV, and the relative bandwidth approximately 10^−4^. The X-ray focus was on the sample membrane with a footprint of about 35(h) × 15(v) *μ*m FWHM, and the transverse coherence length was larger than the size of one particle in both directions. At 7.2 m from the sample position, a Pilatus 2 M detector^40^ was placed after an evacuated flight tube. For each sample, 5151 exposures were taken by scanning the membrane on a rectangular grid. Each exposure lasted one second, with about 10^11^ X-ray photons illuminating the sample.

### Calculation of the angular correlations

The experimental correlations were calculated for each data set *r* as described in ref. 27. The intensity *I*^*r*^(*q, ϕ*) of each Pilatus 2 M pixel image was averaged in bins corresponding to a polar discretization of the 2D reciprocal space. We used 128 sectors in the azimuthal *ϕ*-direction and 160 equally sized sectors in the radial direction in the *q*-range 0.009–0.24 nm^−1^, resulting in a *q*-spacing of 0.0015 nm^−1^ that corresponds to two detector pixels. The Fourier components 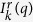 were computed via discrete Fourier transform in the *ϕ*-direction.

### Singular value decomposition

All experimental correlations measured in the *n*_d_ datasets are rearranged in a *n*_m_ × *n*_d_-dimensional real matrix 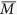 with *n*_m_ > *n*_d_. Its singular value decomposition (SVD) is^11^,^12^


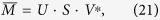


where *U* and *V* are real *n*_m_ × *n*_d_- and *n*_d_ × *n*_d_-dimensional matrices, respectively, while *S* is a real *n*_d_ × *n*_d_-dimensional diagonal matrix with the singular values 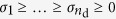 on the diagonal. From their inspection, the number *n*_s_ of relevant components, corresponding to the number of particle species, can be established^11^,^12^. The matrix *S* is then truncated to the matrix 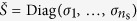, and in parallel *U* and *V* are shrinked to 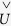 and 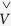 by keeping the first *n*_s_ columns, thus obtaining *n*_m_ × *n*_s_- and *n*_s_ × *n*_s_-dimensional matrices. Up to small errors due to the truncation, the above SVD of 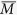 becomes


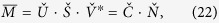


whereby the splitting into the *n*_m_ × *n*_s_- and *n*_s_ × *n*_d_-dimensional matrices 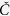 and 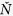 is not unique, as remarked throughout this article. For example, one possibility is to set


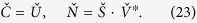


### Search of the disentangling matrix

Let 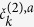 be the 2-point correlation matrices from column *a* of the matrix 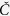 and *A* be a matrix that mediates the transformation (10). Requirement (13) means that for each column index *a* and Fourier order *k* the following must hold:


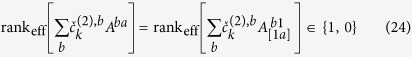


where the matrix *A*_[1a]_ is obtained by permuting the first column with the *a*-th column of the matrix *A*. As stated by Iwasawa^41^, *A*_[1a]_ can then be decomposed as


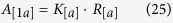


with *K*_[*a*]_ ∈ *SO(n*_s_) an *n*_s_-dimensional orthogonal matrix and *R*_[*a*]_ an upper triangular matrix. It follows that Eq. (24) is equivalent to


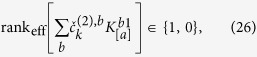


and therefore the parameter search can be restricted to 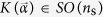, where 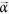 is a collective label for the *n*_s_(*n*_s_ − 1)/2 parameters. In practice, one has to find the *n*_s_ parameter values 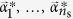 which satisfy the requirement


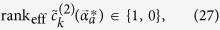


where


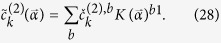


Finally, one sets


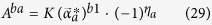


where the sign 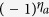 is chosen to ensure that 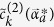 is positive definite. For *n*_s_ = 2, Eq. (28) corresponds to Eq. (16), and Eqs (29) to (17).

### Final renormalization of correlations and populations, single-particle diffraction pattern determination

Let 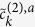 be the 2-point correlation matrices from column *a* of the matrix 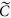, corresponding to the particle species *a*. Moreover, let *D* be the diagonal matrix that mediates the transformation (14), with *δ*^*a*^ the species-specific renormalization parameters on the diagonal entries. From Eqs (6) and (7) it follows that the expression of the single-particle diffraction intensity (1) of species *a* takes the form


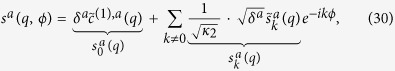


where, for *k* ≠ 0, 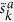 are the eigenvectors to the dominant eigenvalue of the matrix 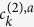 as expressed by Eq. (12). The overall phase of the 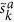 can be determined using the information from the 3-point correlations^27^, which can be disentangled from the experimental values in the different data sets into species-specific values in the same way as done for 1- and 2-point correlations. To asses the value of the parameters *δ*^*a*^, we required the diffraction intensity of each single particle (30) to be positive with isolated zeros. In practice, we increased their value from zero until small regions of negative intensity appeared (see ref. 27). Within this procedure, *κ*_2_ remains an unknown overall beam shape parameter, which we set to the same value *κ*_2_ = 0.25 used in ref. 27. Modification of the parameter *κ*_2_ → *κ*_2_^′^ is equivalent to a global rescaling *C*^*a*^ → *ζ*^−1^ · *C*^*a*^, *S*^(*a*)^ → *ζ*^−1^ · *S*^(*a*)^ and *N*^*a,r*^ → *ζ* · *N*^*a,r*^, with 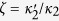.

### Charge density reconstruction by phase retrieval

For the reconstruction of the structures, the obtained expression (1) in polar coordinates (*q, ϕ*) was interpolated onto a Cartesian grid (*q*_*x*_, *q*_*y*_), suitable for the fast Fourier transformation of the phase retrieval algorithm. A rectangular support region of the 2D electron density was determined from the charge density autocorrelation, calculated as the inverse Fourier transform of the single-particle diffraction image. The region served as real-space constraint for the iterative transform algorithm. More precisely, the procedure^42^ consisted of a series of 40 hybrid input-output^43^ (HIO) iterations followed by 10 error-reduction steps, repeated until a total of 1000 iterations was reached. The reconstructions were repeated with 20 different random starting phases, registered within a fraction of a pixel^44^, and averaged to obtain the final 2D structure.

## Additional Information

**How to cite this article:** Pedrini, B. *et al*. Model-independent particle species disentanglement by X-ray cross-correlation scattering. *Sci. Rep.* **7**, 45618; doi: 10.1038/srep45618 (2017).

**Publisher's note:** Springer Nature remains neutral with regard to jurisdictional claims in published maps and institutional affiliations.

## Supplementary Material

Supplementary Figure
