# Supplementary material for: Model-independent particle species disentanglement by X-ray cross-correlation scattering
Source: Sci Rep. 2017 Apr 4;7:45618. doi: 10.1038/srep45618 (PMC5379484; doi:10.1038/srep45618)
Supplement: Supplementary Figure [file srep45618-s1.pdf]

Supplementary material to

Model-independent particle species disentanglement by X-ray cross-correlation scattering.

B. Pedrini[1], A. Menzel[1], V. A. Guzenko[1], C. David[1], R. Abela[1] and C. Gutt[2]

[1] *Paul Scherrer Institute, 5232 Villigen PSI, Switzerland*

[2] *Department Physik, Naturwissenschaftlich-Technische Fakultät, Universität Siegen, 57068 Siegen, Germany*

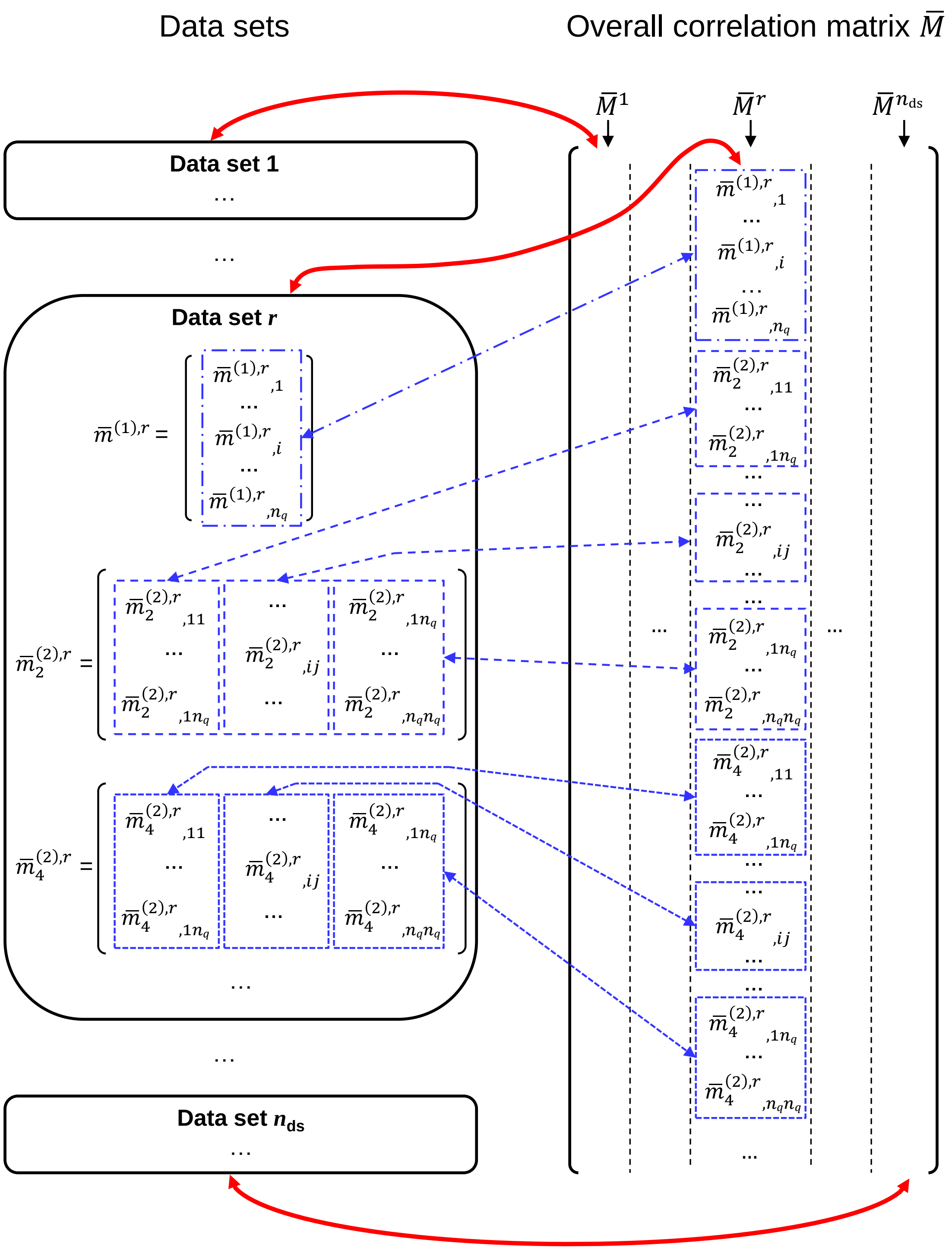

Supplementary Figure S1. *Overall correlation matrix* $\bar{M}$. Description: see next page.

Supplementary Figure S1. *Overall correlation matrix $\bar{M}$.*

The figure illustrates in detail how all experimental correlations (on the left) are rearranged into the overall correlation matrix $\bar{M}$ (on the right). Each column $\bar{M}^r$ corresponds to the data set labeled by r (see red arrows). The blue arrows indicate the exact correspondence between the column entries and the correlations. The correlations are assumed to be determined from the experimental data at discrete values of momentum transfer $q_1..q_{n_q}$. Therefore the 1-point correlations are arranged in an n_q-dimensional column vector $\bar{m}^{(1),r}{}_{,i}$, occuping n_q entries of the column $\bar{M}^r$, while the for each Fourier order k the 2-point correlations are arranged in an $n_q \times n_q$-dimensional matrix $\bar{m}_k^{(2),r}{}_{,ij}$, which each occupy $n_q \times n_q$ entries of the same column.

A totally equivalent correspondence applies between single particle correlations $c^{(1),r}{}_{,i}$ and $c_k^{(2),r}{}_{,ij}$ of species a, and the columns C^a of the overall single particle correlation matrix C.
